# Supplementary material for: Inflammatory factors and risk of lung adenocarcinoma: a Mendelian randomization study mediated by blood metabolites
Source: Front Endocrinol (Lausanne). 2024 Aug 27;15:1446863. doi: 10.3389/fendo.2024.1446863 (PMC11384989; doi:10.3389/fendo.2024.1446863)
Supplement: Supplementary file 1 [file DataSheet1.zip › supplementary files/S2/GCST90200768/forest.pdf]

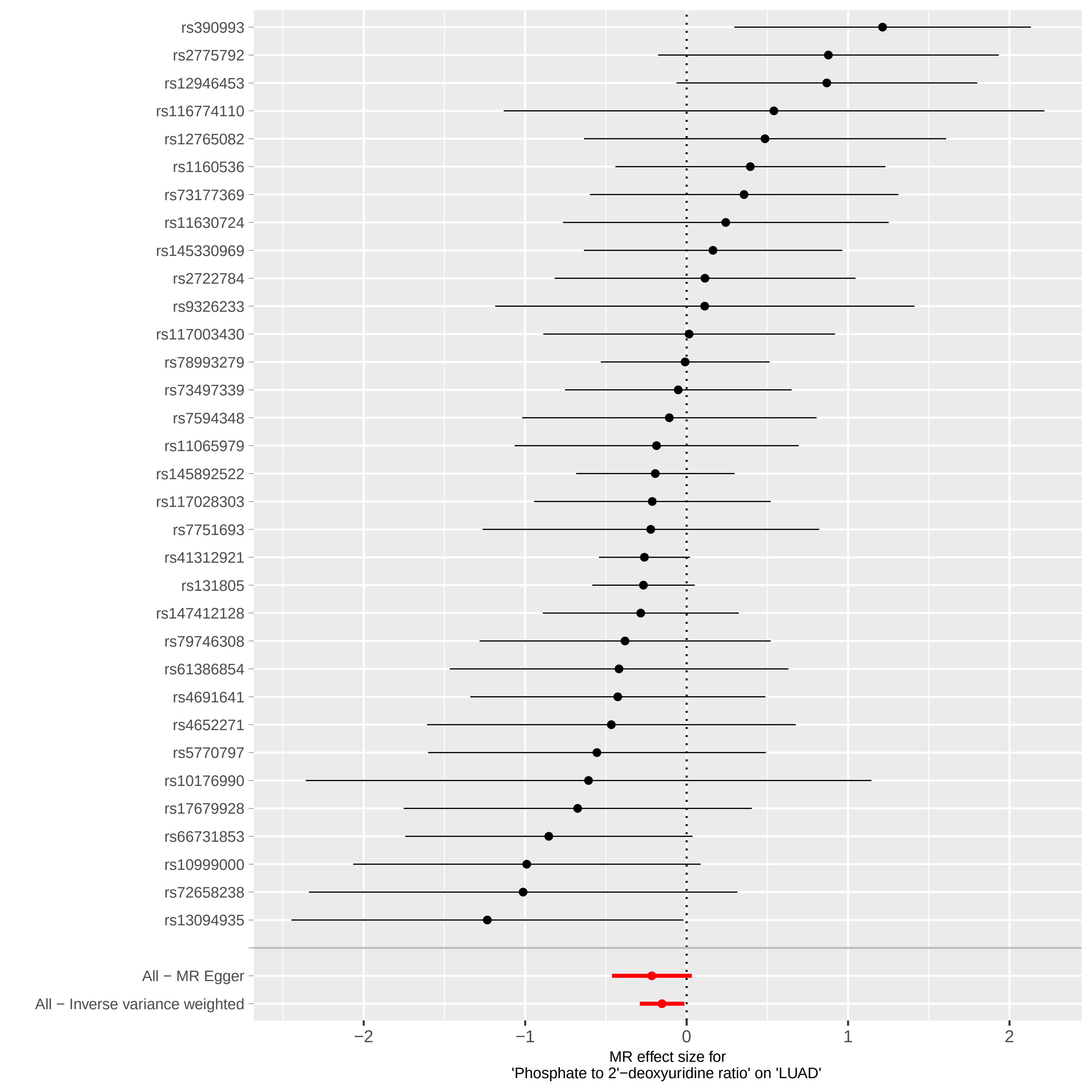
rs390993
rs2775792
rs12946453
rs116774110
rs12765082
rs1160536
rs73177369
rs11630724
rs145330969
rs2722784
rs9326233
rs117003430
rs78993279
rs73497339
rs7594348
rs11065979
rs145892522
rs117028303
rs7751693
rs41312921
rs131805
rs147412128
rs79746308
rs61386854
rs4691641
rs4652271
rs5770797
rs10176990
rs17679928
rs66731853
rs10999000
rs72658238
rs13094935
All − MR Egger
All − Inverse variance weighted
−2
−1
0
1
2
MR effect size for
'Phosphate to 2'−deoxyuridine ratio' on 'LUAD'
